# Visceral Adipose Tissue Bioenergetics Varies According to Individuals’ Obesity Class

**DOI:** 10.3390/ijms24021679

**Published:** 2023-01-14

**Authors:** Marcelo V. Topete, Sara Andrade, Raquel L. Bernardino, Marta Guimarães, Ana M. Pereira, Sofia B. Oliveira, Madalena M. Costa, Mário Nora, Mariana P. Monteiro, Sofia S. Pereira

**Affiliations:** 1 UMIB-Unit for Multidisciplinary Research in Biomedicine, ICBAS-School of Medicine and Biomedical Sciences, University of Porto, Rua Jorge Viterbo Ferreira 228, 4050-313 Porto, Portugal; 2 ITR-Laboratory of Integrative and Translocation Research in Population Health, Rua das Taipas 135, 4050-600 Porto, Portugal; 3 Department of General Surgery, Hospital São Sebastião, Centro Hospitalar de Entre o Douro e Vouga, Rua Dr. Cândido Pinho, 4050-220 Santa Maia da Feira, Portugal; 4 Hospital da Luz Arrábida, Praceta de Henrique Moreira 150, 4400-346 Vila Nova de Gaia, Portugal

**Keywords:** obesity, white adipose tissue, mitochondrial dysfunction, pyruvate, glutamine, fatty acids

## Abstract

Obesity is associated with complex adipose tissue energy metabolism remodeling. Whether AT metabolic reprogramming differs according to body mass index (BMI) and across different obesity classes is unknown. This study’s purpose was to evaluate and compare bioenergetics and energy substrate preference of visceral adipose tissue (VAT) pertaining to individuals with obesity class 2 and class 3. VAT obtained from patients with obesity (n = 15) class 2 (n = 7; BMI 37.53 ± 0.58 kg/m^2^) or class 3 (n = 8; BMI 47.79 ± 1.52 kg/m^2^) was used to assess oxygen consumption rate (OCR) bioenergetics and mitochondrial substrate preferences. VAT of patients with obesity class 3 presented significantly higher non-mitochondrial oxygen consumption (*p* < 0.05). In VAT of patients with obesity class 2, inhibition of pyruvate and glutamine metabolism significantly decreased maximal respiration and spare respiratory capacity (*p* < 0.05), while pyruvate and fatty acid metabolism inhibition, which renders glutamine the only available substrate, increased the proton leak with a protective role against oxidative stress (*p* < 0.05). In conclusion, VAT bioenergetics of patients with obesity class 2 depicts a greater dependence on glucose/pyruvate and glutamine metabolism, suggesting that patients within this BMI range are more likely to be responsive to interventions based on energetic substrate modulation for obesity treatment.

## 1. Introduction

Adipose tissue (AT) is one of the largest organs in the human body. Obesity is characterized by abnormal AT deposition with adipocyte expansion in several anatomical locations, including subcutaneous and visceral territories, such as the intra-abdominal region [1,2,3]. Visceral adipose tissue (VAT), in addition to storing excess energy in the form of fat, also plays important energy regulatory functions [2,4]. Imbalances in substrate availability can lead to mitochondrial dysfunction, with potential impacts on energy production and oxidative respiration [5]. Despite adipocyte mitochondrial density being relatively low, these organelles have an essential role in different cellular metabolic pathways [5]. Therefore, mitochondrial dysfunction inevitably influences the adipocyte dynamics by altering adipogenesis and lipogenic and lipolytic pathways [5,6]. In individuals with obesity, adipocytes are characterized by presenting mitochondria with ill-defined inner membranes and lower energy-generating and reduced fatty acid oxidation capacities, when compared to those of individuals without obesity [5,7,8,9]. Moreover, adipocytes tend to divert glucose and fatty acid overload towards acetyl-CoA production, which in turn increases electron availability for mitochondrial respiratory chain complexes, which leads to reactive oxygen species (ROS) production [10,11]. ROS-induced oxidative stress then triggers the activation of transcription factors involved in the inflammatory response often present in obesity [8,11,12]. In addition, AT expansion can also lead to oxygen deprivation and hypoxia, further impairing mitochondrial oxidative metabolism and, thus, contributing to the perpetuation of mechanisms involved in inflammation [13,14,15]. Although VAT mitochondrial dysfunction has been well-documented in individuals with obesity when compared with normal-weight individuals, how VAT mitochondrial function varies across obesity classes is still poorly understood.

## 2. Results

### 2.1. Subjects’ Demographics and Anthropometric Characteristics

Female patients (n = 15) with an average age of 44.93 ± 3.17 years (range: 23–62 years) and a BMI of 43.00 ± 1,60 kg/m^2^ (ranging from 35.40–54.40 kg/m^2^) were allocated into two study groups according to obesity class. Patients presented an average fasting plasma glucose of 98.40 ± 3.62 mg/dL and an average glycated hemoglobin (HbA1c) value of 5.56 ± 0.10. The systemic inflammatory status of the patients was also accessed by the neutrophil-to-lymphocyte ratio (NLR) and platelet-to-lymphocyte ratio (PLR). Subjects presented an average NLR of 1.87 ± 0.18 and an average PLR of 5.56 ± 0.10. Subjects’ characteristics are presented in Table 1.

### 2.2. Bioenergetics Assays

Total basal oxygen consumption, mitochondrial basal respiration, non-mitochondrial oxygen consumption, maximum respiration, spare respiratory capacity, proton leak and ATP-linked respiration were evaluated by measuring the oxygen consumption rate (OCR) under the influence of metabolic pathways inhibitors and compared in VAT pertaining to patients with obesity class 2 and class 3. 

#### 2.2.1. Obesity Class 3 Presents Higher Non-Mitochondrial Oxygen Consumption 

**Figure 1:**
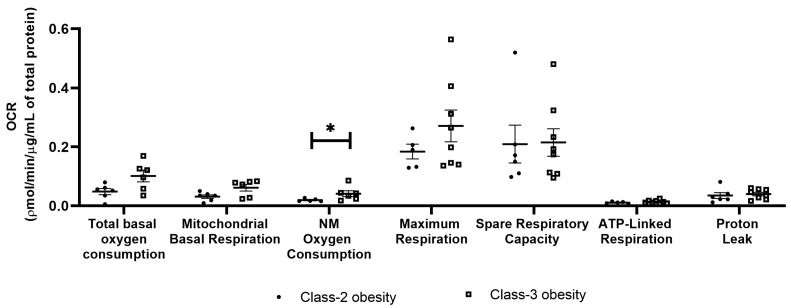
Comparison of VAT respiratory parameters of patients with obesity class 2 or 3 (Mann–Whitney test * *p* < 0.05). OCR—Oxygen Consumption Rate; NM—Non-mitochondrial.

Non-mitochondrial oxygen consumption was higher in VAT of patients with obesity class 3 when compared to VAT of patients with obesity class 2 (0.04 ± 0.01 pmol/min/μg/mL of total protein vs. 0.02 ± 0.002 pmol/min/μg/mL of total protein, *p* < 0.05). It is worth noting that although VAT of patients with obesity class 3 presented numerically higher total oxygen consumption, mitochondrial respiration and maximum respiration, no statistically significant differences were identified between the two patient groups (Figure 1).

#### 2.2.2. Under Conditions of Higher Energy Demands, VAT OCR Depends on Pyruvate Metabolism 

**Figure 2:**
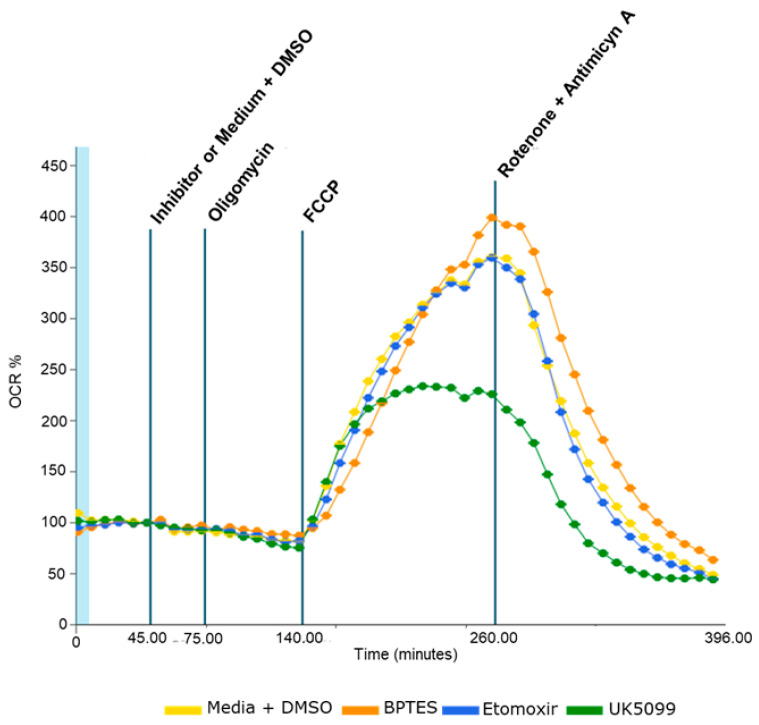
Oxygen consumption rate (OCR) during the bioenergetics assay. Inhibitors of the pyruvate carrier (UK5099), glutaminase 1 (BPTES) and carnitine palmitoyltransferase 1 (Etomoxir) were used to evaluate the contribution of pyruvate, glutamine and fatty acid metabolism, respectively, for VAT OCR. DMSO with no inhibitor was used as control. Oligomycin (an ATP synthetase inhibitor), carbonyl cyanide p-trifluoromethoxyphenylhydrazone (FCCP, a mitochondrial oxidative phosphorylation uncoupler) and a combination of rotenone and antimycin A (mitochondrial complexes 1 and 3 inhibitors) were added in order to modulate the electron transport chain.

Regardless obesity class, the inhibition of pyruvate, glutamine or fatty acid metabolism does not affect VAT baseline OCR. However, under the influence of the carbonyl cyanide p-trifluoromethoxyphenylhydrazone (FCCP), a mitochondrial oxidative phosphorylation uncoupler that allows a rapid translocation of protons across the inner mitochondrial membrane, mimicking a high energy demand condition, pyruvate metabolism inhibition with a pyruvate carrier inhibitor (UK5099) suppressed the OCR, which did not occur after glutamine or fatty acid metabolism inhibition (Figure 2).

#### 2.2.3. In Obesity Class 2, VAT Maximal Respiration and Spare Respiratory Capacity Are Dependent on Pyruvate and Glutamine Metabolism 

**Figure 3:**
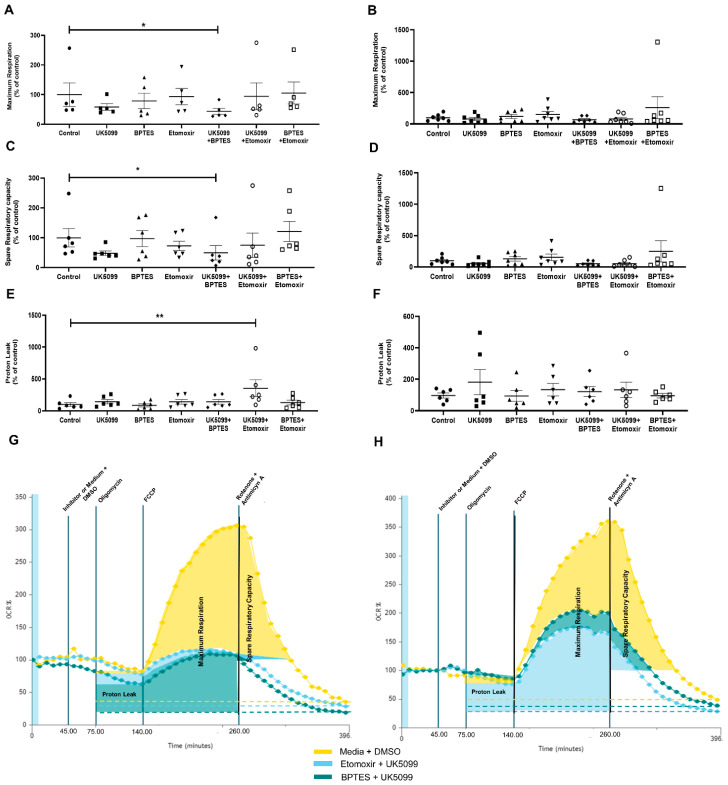
Influence of metabolic pathway inhibitors on maximum respiration, spare respiratory capacity and proton leak of VAT pertaining to patients with obesity class 2 (**A**,**C**,**E**) or class 3 (**B**,**D**,**F**) (Friedman Test: * *p* < 0.05, ** *p* < 0.01). Illustrative graphics of VAT OCR vs. Time from patients with obesity class 2 (**G**) or 3 (**H**) obtained by Wave Software (Agilent Technologies). Colored areas represent different metabolic parameters that were calculated.

Inhibition of pyruvate and glutamine metabolism, by the combination of pyruvate carrier (UK5099) and glutaminase 1 (BPTES) inhibitors, was associated with significantly lower VAT maximal respiration and spare respiratory capacity (maximum respiration: 43.94 ± 10.49% vs. 100.00 ± 39.57%, *p* < 0.05; spare respiratory capacity: 49.81 ± 24.17% vs. 100.00 ± 30.62%, *p* < 0.05, Figure 3A,C and Figure S1). However, these differences were only significant for VAT pertaining to patients with obesity class 2 (Figure 3B,D and Figure S2).

#### 2.2.4. In Obesity Class 2, VAT Proton Leak Is Positively Influenced by Glutamine Metabolism 

Pyruvate and fatty acid metabolism inhibition, by the combination of pyruvate carrier (UK5099) and carnitine palmitoyltransferase 1 inhibitors (Etomoxir), which renders glutamine the only available substrate, was associated with a significantly higher VAT proton leak compared to baseline conditions (355.50 ± 131.90% vs. 100.00 ± 28.58%, *p* < 0.01, Figure 3E), although this effect was only significantly for VAT pertaining to patients with obesity class 2 (Figure 3E and Figure S2).

#### 2.2.5. VAT Bioenergetics Parameters Do Not Correlate with Age 

Since patients enrolled in this study presented a wide age range (Δ 41 years; 23–62 years), we sought to analyze whether VAT bioenergetics could have been influenced by age through testing for the presence of significant correlations between age and the different calculated parameters. No significant correlations were identified (Table S1).

### 2.3. In Obesity Classes 2 and 3, VAT Expression of Genes Involved in Pyruvate, Glutamine and Fatty Acid Metabolism Does Not Differ

**Figure 4:**
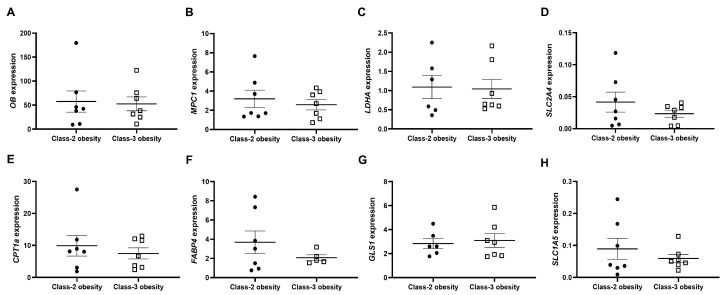
Expression of (**A**) *OB*; (**B**) *MPC1*; (**C**) *LDHA*; (**D**) *SLC2A4*; (**E**) *CPT1a*; (**F**) *FABP4*; (**G**) *GLS1* and (**H**) *SLC1A5* genes in VAT pertaining to patients with obesity classes 2 and 3. (*OB*—Leptin; *MPC1*—Mitochondrial Pyruvate Carrier 1; *LDHA*—Lactate Dehydrogenase A; *SLC2A4*—GLUT4; *CPT1a*—Carnitine Palmitoyltransferase 1A; *FABP4*—Fatty Acid-Binding Protein 4; *GLS1*—Glutaminase 1; *SLC1A5*—Neutral amino acid transporter). Results were normalized for the expression of two housekeeping genes (*RPII* and *β-actin*).

To evaluate whether the bioenergetics differences identified in VAT of patients with obesity classes 2 and 3 could have been influenced by the number of adipocytes per mass of VAT or the expression of genes involved in pyruvate, glutamine, and fatty acid metabolism, *OB, MPC1*, *LDHA*, *SLC2A4*, *CPT1a*, *FABP4*, *GLS1* and *SLC1A5* gene expressions were evaluated. No significant differences in gene expression between VAT of patients with obesity classes 2 and 3 were observed (Figure 4).

## 3. Discussion

Bioenergetics corresponds to the energy flow through living systems. Unveiling bioenergetics dynamics is essential for understanding the mechanisms underlying energy balance disorders, identifying biomarkers of disease progression and pinpointing molecular targets that could lead to new treatment strategies. The development of novel equipment for bioenergetics analysis (e.g., extracellular flux analysis, high-resolution respirometry, etc.) has enabled the rapid assessment of several metabolic parameters [16,17]. Mitochondria are organelles that respond dynamically to changes within their microenvironment and are very susceptible to stress [16]. Obesity-associated adipose tissue mitochondrial dysfunction has been previously described and widely confirmed [5,6,8,13]. However, whether VAT mitochondrial dysfunction differs according to BMI and across different obesity classes is unknown. Thus, the aim of this study was to evaluate and compare bioenergetics and energy substrate preference of VAT pertaining to individuals with obesity class 2 and class 3. Toward this aim, VAT harvested during elective surgical procedures was incubated with specific inhibitors of the three main mitochondrial energy substrates: glutamine, pyruvate and fatty acids, and under the influence of electron transport chain (ETC) modulators.

Our study found that non-mitochondrial oxygen consumption was higher in VAT of patients with obesity class 3 when compared to those of patients with obesity class 2. Obesity is associated with higher production of ROS and excessive accumulation of pro-inflammatory macrophages within the VAT, a phenomenon that characterizes a chronic low-grade inflammatory status [14,18,19]. Thus, the greater non-mitochondrial oxygen consumption observed in VAT of patients with obesity class 3 could result from the activity of pro-oxidant and pro-inflammatory enzymes, such as cyclo-oxygenases, cytochrome P450 or NADPH oxidases, suggesting a greater inflammatory activity [16]. Although we did not find any significant differences in the systemic inflammatory response markers (NLR and PRL) between the groups, we cannot exclude the notion that differences might exist in local VAT inflammation. In the future, pro-oxidant and inflammatory markers in VAT of patients with obesity class 2 or 3 would be important to validate this hypothesis. 

In addition, previous in vitro and in vivo studies sought to evaluate the putative efficacy of antioxidant drugs and bioenergetics modulators for obesity treatment [20,21,22,23]. Since the VAT samples of patients with class-2 and class-3 obesity have different bioenergetic profiles, the impact of antioxidant drugs can also be different and should be addressed in future studies. 

VAT basal respiration of patients with obesity was not influenced by any of the substrate’s inhibitors, supporting previous data demonstrating that adipose tissue presents a high degree of metabolic plasticity [24]. However, in the present study, we show that under conditions of greater energy demands, pyruvate is the VAT-preferential mitochondrial substrate, since the maximum respiration and the percentage of spare capacity are decreased when the entrance of pyruvate into the mitochondria is blocked by MPC1 inhibition. This phenomenon became particularly evident when both pyruvate and glutamine metabolisms were inhibited. Once again, our findings are further supported by previous studies which demonstrated that glutamine can be used to compensate glucose/pyruvate metabolism inhibition [25]. Therefore, although adipocytes are reservoirs of fatty acids, these substrates are not used as mitochondrial substrates, not even in conditions of higher energy demands or shortages of preferential substrates, namely pyruvate and glutamine. Interestingly, the decrease in maximum respiration and percentage of spare capacity after pyruvate and glutamine metabolism inhibition is a specific characteristic of VAT of patients with obesity class 2. Although these data need to be validated in larger patient groups, these findings still suggest that the VAT of patients with obesity class 3 depicts a greater metabolic plasticity under higher energy demand conditions, being able to adapt its metabolism to use whichever substrate is available. It is worth mentioning that the class-2 obesity group, in which the significant differences were observed, had a smaller number of subjects than the group with obesity class 3, which renders a statistical type II error less likely.

In subjects with obesity class 2, the VAT proton leak was higher when glutamine was the only mitochondrial energetic substrate available. Glutamine is a non-essential amino acid that can be used to obtain energy through glutaminolysis. This pathway corresponds to the conversion of glutamine into glutamate to produce key metabolic intermediaries for fatty acid biosynthesis and to feed the tri-carboxylic acid (TCA) cycle [26,27]. Glutamine was previously shown to inhibit glucose-6-fostate and 3-fosfoglycerol, reducing glycolysis and increasing TCA cycle activity, which leads to a higher rate of NADH and FADH_2_ generation, used as electron carriers in ETC [25]. Higher oxygen consumption by ETC results in greater production of ROS and, consequently, to a rapid depolarization of the inner membrane potential. Proton leak is a mitochondrial protective mechanism to decrease ROS production [28,29]. Since lipid membranes have a high conductance, these allow protons to migrate across the inner membrane independently of complex V, i.e., proton leak [29]. There are two forms of proton leak regulation: constitutive and inducible. Constitutive proton leakage regulation depends on the phospholipids fatty acid composition of the inner membrane. Inducible proton leak regulation depends on the inner membrane uncoupling proteins (UCPs) [29,30]. The UCPs are regulated at the molecular, transcriptional, translational and proteolytic levels, and several studies suggest its involvement in cardiovascular disease, oxidative stress, type 2 diabetes (T2D) and immune response [29,31,32]. UCP-2 is ubiquitously expressed in most cell types and tissues, while UCP-3 is less abundantly expressed [33]. UCP-1 is the most studied UCP protein, since it was shown to be present in BAT and associated with non-shivering thermogenesis [34]. Obesity is associated with lower VAT UCP-1, -2 and -3 expression [35,36]. Glutamine was found to increase UCP-2 expression in the inner membrane of neuroblastoma cells, as a mechanism of defense against ROS [37]. Thus, we postulate that a similar mechanism might occur in the VAT of patients with obesity class 2, since an increase in proton leak was observed in the presence of an active glutamine metabolism. Glutamine levels were found to be decreased in obesity, while glutamine deficiency was appointed as playing a role in obesity-associated inflammation once this amino acid attenuates VAT expression of pro-inflammatory genes/proteins [25]. However, this mechanism seems to be hampered in patients with obesity class 3; when glutamine was the only energetic substrate available, there was no significant increase in proton leak, contrary to what was observed in the VAT of patients with obesity class 2.

Mitochondrial dysfunction is a hallmark of aging [38], which is associated with a decrease in ETC complex IV activity [39]. Nonetheless, in our study, age did not seem to have a significant impact on VAT oxygen consumption or bioenergetics parameters. This could be attributed to the fact that obesity per se is associated with considerable mitochondrial dysfunction, so age does not depict significant additional effects.

In this study, we decided to analyze whole VAT bioenergetics instead of isolated adipocytes in an attempt to mimic ex vivo the in vivo state. Moreover, *OB* gene expression was used as a surrogate measure of the number of adipocytes in each VAT fragment, since this gene is primarily expressed by mature adipocytes [40,41]. We found that VAT *OB* gene expression was not significantly different between obesity classes and so the bioenergetics results do not seem to have been influenced by different adipocyte cell number within the analyzed VAT cell mass. In addition, bioenergetics assays involved blocking specific proteins that participate in pyruvate, glutamine and fatty acid metabolism. The expression of some of these genes, namely *CPT1* [42], *SLC2A4* [43] and *MCP1* [44], were previously shown to be downregulated in the VAT of individuals with obesity. In order to evaluate whether the differences observed could have been attributed to the fact that these genes were differentially expressed across obesity classes, gene expression was also analyzed. No significant differences in these genes’ expression were observed in the VAT of subjects with obesity class 2 and class 3, which could have influenced the bioenergetics. It is important to stress that bioenergetics profiles of other VAT cell types should also have been assessed (e.g., macrophage populations) in order to evaluate their relative contribution for our results. However, the limited size of the VAT sample did not allow us to perform any further analysis. 

## 4. Materials and Methods

### 4.1. Subjects and Samples Collection

Patients planning to undergo bariatric surgery for the primary treatment of obesity were invited to participate in this study. Subjects with T2D, a condition known to be associated with mitochondrial dysfunction, or prior history of neoplastic disease or other bariatric surgery intervention were excluded. Patients were stratified according to BMI and allocated into two study groups: class-2 obesity (BMI between 35 and 40 kg/m^2^, n = 7), and class-3 obesity (BMI ≥ 40 kg/m^2^, n = 8).

Preoperative blood sampling performed as part of routine clinical assessment was used to perform full blood count at the hospital laboratory. NLR and PLR were then calculated as systemic inflammatory response (SIR) markers. NLR and PLR were determined by dividing the absolute neutrophil count by the absolute lymphocyte count and by dividing the absolute platelet count by the absolute lymphocyte count, respectively. A NLR value above 3.0 and a PLR of 150 were considered pathological [45].

### 4.2. VAT Bioenergetic Analysis

#### 4.2.1. Oxygen Consumption Rate Assessment

Agilent Seahorse XF technology was used to evaluate mitochondrial respiration through the measurement of real-time tissue OCR. On the day before surgery and planned VAT harvesting, the sensor cartridge was hydrated with a commercial calibration solution (XF Calibrant pH 7.4, Agilent Technologies, Santa Clara, CA, USA) at 37 °C in a CO_2_-free incubator. On the following day, VAT was minced into small fragments (~5 mg) to be placed and attached to the bottom of an assay plate well by capture screens, before being incubated in culture medium (Seahorse XF DMEM Medium, pH 7.4, 103575-100, Agilent Technologies). After that, VAT was incubated with the assay medium supplemented with 10 mM glucose (103577-100, Agilent Technologies), 2 mM glutamine (103579-100, Agilent Technologies), 0.17 mM of sodium palmitate (P9767-5G, Sigma-Aldrich, St. Louis, MO, USA) and 0.5 mM of L-carnitine (C0283-1G, Sigma-Aldrich) before being placed in the analyzer (Seahorse XFe24 Analyzer, Agilent Technologies). After an initial period of equilibration, metabolic pathway inhibitors and ETC modulators were automatically added into the cartridge wells according to the following sequential order: Firstly, different metabolic pathway inhibitors were added—the mitochondrial pyruvate carrier inhibitor UK5099 (final concentration of 4 μM (5.04817.0001, EMD Millipore Corporation, USA)), the glutaminase 1 inhibitor BPTES, ((bis-2-(5-phenylacetamido-1,3,4-thiadiazol-2-yl)ethyl sulfide, final concentration of 6 μM, SLM0601, Sigma-Aldrich) and the inhibitor of carnitine palmitoyltransferase-1, Etomoxir (final concentration of 8 μM; 236020, EMD Millipore Corporation). During the assay, these solutions were added singly or in combinations into the wells containing VAT fragments in triplicates. Medium with dimethyl sulfoxide (DMSO, final concentration of 0.2%; D2650-5X10ML, Sigma-Aldrich) was used as control. Afterwards, ETC modulators were added, starting with an ATP synthetase inhibitor oligomycin (final concentration of 12.5 μg/mL; O4876, Sigma-Aldrich), followed by a mitochondrial oxidative phosphorylation uncoupler FCCP (final concentration of 8 µM; C2920, Sigma-Aldrich), and ending with the addition of a combination of mitochondrial complexes 1 and 3 inhibitors rotenone (final concentration of 2 µM; R8875, Sigma-Aldrich) and antimycin A (final concentration of 4 μM, A8674, Sigma-Aldrich). 

#### 4.2.2. Total Protein Extraction and Quantification for OCR Normalization 

At the end of the bioenergetic assay, VAT fragments were retrieved from the assay plate well for total protein extraction using a protocol previously optimized for adipose tissue [46]. For that, VAT fragments were incubated overnight at 4 °C in RIPA buffer (RO278-500 ML, Sigma-Aldrich) supplemented with protease (04693124001, Roche, Basel, Switzerland) and phosphatase inhibitors (04906845001, Roche, Switzerland). Afterwards, VAT fragments were lysed using a FastPrep^®^-24 Instrument (MP biomedicals, Irvine, CA, USA) at a speed of 6.0 m/s for 30 s. VAT lysates were then incubated on ice for 1 h and centrifuged at 20,000× *g* for 15 min at 4 °C. Proteins were quantified using the commercial Pierce™ BCA Protein Assay Kit (23225, Thermo Fisher Scientific, Rockford, IL, USA). Briefly, bovine serum albumin (BSA) standards (0 to 2000 µg/mL) and the working reagent (WR) were prepared. In a 96-well plate, 25 µL of each sample or the BSA standard and 200 µL of WR reagent were added to each well, in duplicate. The plate was incubated for 30 min at 60 °C in a dark room. Finally, the absorbance (560 nm) was read on the microplate reader (Thermo Scientific™ Multiskan™ FC Microplate Photometer, Thermo Fisher Scientific, Rockford, IL, USA). Protein quantification results were used for OCR normalization. 

#### 4.2.3. Bioenergetic Parameter Calculations

Seahorse Wave 2.6.3. software was used for analysis and calculation of the following parameters: total oxygen consumption; non-mitochondrial oxygen consumption; mitochondrial respiration; ATP-linked respiration; maximum respiration; spare respiratory capacity; proton leak and acute response to inhibitors. The calculation formulas are depicted in Table S2.

### 4.3. Gene Expression Analysis

#### 4.3.1. RNA Isolation and Quantification

Total RNA was extracted from VAT (~100 mg) homogenized with the aid of a TissueRuptor^®^ (QIAGEN, Hilden, Germany), using the RNeasy^®^ Lipid Tissue kit (74804, QIAGEN, Hilden, Germany) following the manufacturer’s instructions. Total RNA then was quantified using a BioTek^®^ SYNERGY^H1^ microplate reader (BioTek^®^, Agilent Instruments, Winooski, VT, USA) with Gen5^TM^ Data Analysis Software (BioTek^®^, Agilent Instruments, Winooski, VT, USA). 

#### 4.3.2. cDNA Synthesis

cDNA was obtained using the Maxima First Strand cDNA Synthesis Kit with double-strand-specific DNases (dsDNase) (K1671, Thermo Fisher Scientific Inc, Rockford, IL, USA USA). Briefly, RNA (250 ng) was incubated with dsDNases in order to remove genomic DNA. After that, the 5× Reaction Mix and the Maxima Enzyme Mix were added. The mixtures were then incubated in a T100™ Thermal Cycler (Bio-Rad, Hercules, CA, USA) using the following program: 10 min at 25 °C followed by 15 min at 50 °C and 5 min at 85 °C.

#### 4.3.3. Primers

The primers for RNA polymerase II (*RPII*), *β-actin*, leptin (*OB*), GLUT4 (*SLC2A4*), mitochondrial pyruvate carrier 1 (*MPC1*), neutral amino acid transporter (*SLC1A5*), glutaminase 1 (*GLS1*) and carnitine palmitoyltransferase 1a (*CPT1a*), were chosen based on previously published studies [47,48,49,50,51,52,53]. The remaining primers, namely *LDHA* and *FABP4*, were designed using the Primer Blast (National Library of Medicine). Primer sequences are shown in Table S3. The housekeeping genes (*RPII* and *β-actin*) were chosen according to previously validated genes for qRT-PCR analysis in human VAT [47].

#### 4.3.4. Quantitative Real-Time Polymerase Chain Reaction Analysis

Quantification of *OB, CPT1a, MPC1, SLC1A5, GLS1, SLC2A4, LDHA, FABP4* and housekeeping genes *β-actin* and *RPII* expression was achieved by quantitative real-time polymerase chain reaction (qRT-PCR) analysis, following the instructions given by NZYSpeedy qPCR Green Master Mix (2×) (MB22402, NZYTech, Lisbon, Portugal). No cDNA was added in the negative control. A duplicate was performed for each sample. After mix preparation, samples were placed in the CFX Connect™ Real-Time System (Bio-Rad, Hercules, CA, USA). The three-step cycling qRT-PCR program was performed as described in Table S4. The quantitative analysis was followed and obtained with the software CFX-Maestro™ (Bio-Rad, Hercules, CA, USA). The qRT-PCR data analysis was conducted using the comparative CT method. Individual relative gene expression values (fold change) were calculated using the following formula: 2^−ΔCt^.

### 4.4. Statistical Analysis

The statistical analysis was performed with GraphPad^®^ Prism software (USA), version 9.5.0 for Windows. Qualitative and quantitative variables are expressed as percentage (%) and mean ± standard error of the mean, respectively. The normality of the variables was evaluated using the D’Agostino–Pearson test. An unpaired Student’s t-test was used for comparison of variables that followed a normal distribution and the Mann–Whitney test for variables without a normal distribution. For the inhibitor analysis, the groups were compared using the Friedman test. The Pearson test or Spearman rank test were used to assess the correlation between two different variables, depending on whether the distribution was normal or non-normal, respectively. *p* < 0.05 was considered statistically significant.

## 5. Conclusions

VAT of patients with class-3 obesity present greater non-mitochondrial respiration when compared to VAT of patients with class-2 obesity, suggesting higher pro-oxidant enzymes and inflammatory activity.

In the VAT of patients with obesity class 2, maximum respiration and percentage of spare capacity are highly dependent on pyruvate and glutamine metabolism, while the VAT of patients with obesity class 3 seems to be able to adapt its metabolism to use whichever substrate is available at a given moment. Moreover, glutamine, by increasing proton leak, seems to have a protective role against ROS production in VAT of patients with obesity class 2, a mechanism that seems to be lost in patients with obesity class 3.

Overall, our results suggest that patients with obesity class 2 are likely to be more responsive to interventions based on energetic substrate availability modulation (as a strategy for the treatment for obesity) than patients with a more advanced obesity class.

## Figures and Tables

**Table 1:** Study subjects’ characteristics.

	Total	Obesity Class 2	Obesity Class 3	*p*-Value
Subjects (n)	15	7	8	-
Age (years)	44.93 ± 3.17	45.57 ± 4.85	44.38 ± 4.48	ns
Weight (kg)	108.10 ± 3.83	99.00 ± 3.37	117.10 ± 4.99	*p* < 0.01
BMI (kg/m^2^)	43.00 ± 1.60	37.53 ± 0.58	47.79 ± 1.52	*p* < 0.001
FPG (mg/dL)	98.40 ± 3.62	100.10 ± 4.2	96.90 ± 5.90	ns
HbA1c (%)	5.56 ± 0.10	5.49 ± 0.14	5.63 ± 0.14	ns
NLR	1.87 ± 0.18	2.10 ± 0.22	1.67 ± 0.26	ns
PLR	5.56 ± 0.10	5.49 ± 0.14	5.63 ± 0.14	ns

The values are presented as mean ± standard error of the mean. BMI—Body Mass Index; FPG—fasting plasma glucose; HbA1c—glycated hemoglobin; NLR—Neutrophil-to-lymphocyte ratio; PLR—platelet-to-lymphocyte ratio.

## Data Availability

The data presented in this study are available upon request from the corresponding author due to ethical concerns.

## Supplementary Materials

The following supporting information can be downloaded at: https://www.mdpi.com/article/10.3390/ijms24021679/s1.
