# Supplementary material for: Visceral Adipose Tissue Bioenergetics Varies According to Individuals’ Obesity Class
Source: Int J Mol Sci. 2023 Jan 14;24(2):1679. doi: 10.3390/ijms24021679 (PMC9863201; doi:10.3390/ijms24021679)
Supplement: Supplementary file 1 [file ijms-24-01679-s001.zip › ijms-2168050-supplementary.pdf]

Visceral adipose tissue bioenergetic varies according to individuals' obesity class

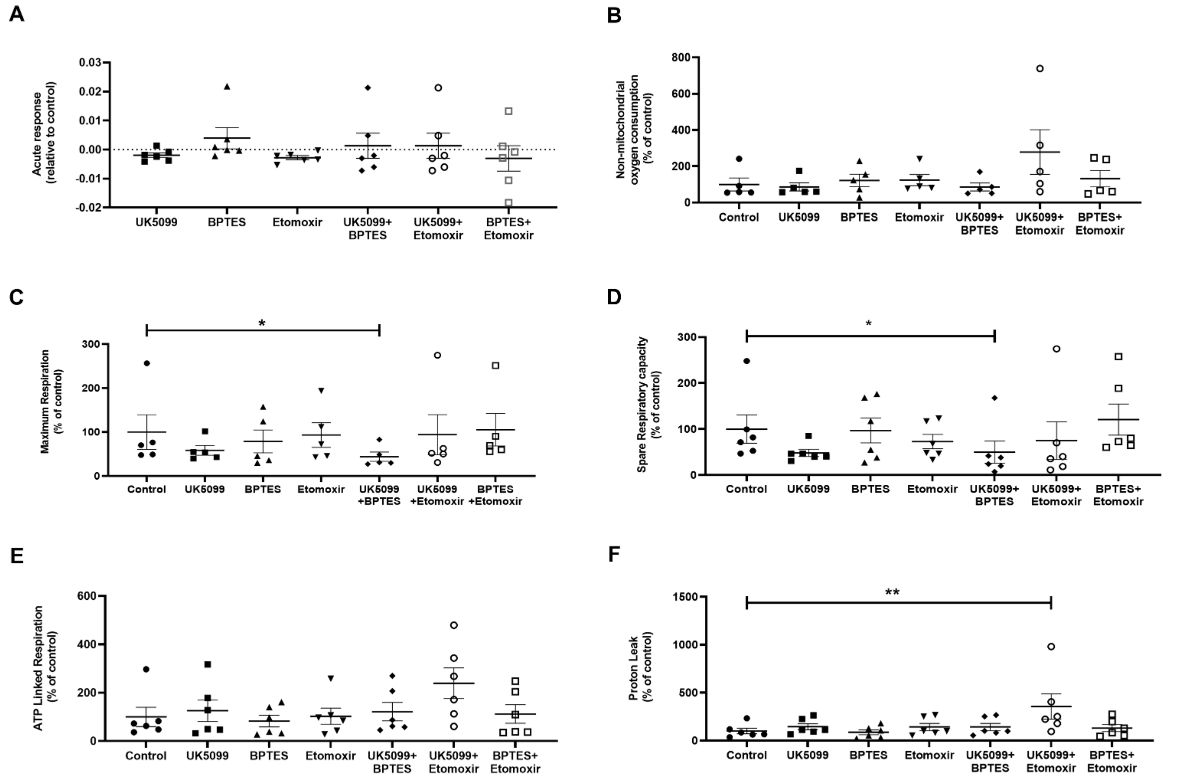

Figure S1. Influence of the inhibitors in (A) basal respiration (acute response); (B) non-mitochondrial oxygen consumption; (C) Maximum respiration oxygen consumption; (D) Spare respiratory capacity; (E) Proton Leak and (F) ATP link respiration in the VAT of patients with a class 2 obesity. Except for the acute response, all values are normalized to the control group. (Friedman test *p<0.05, **p<0.01).

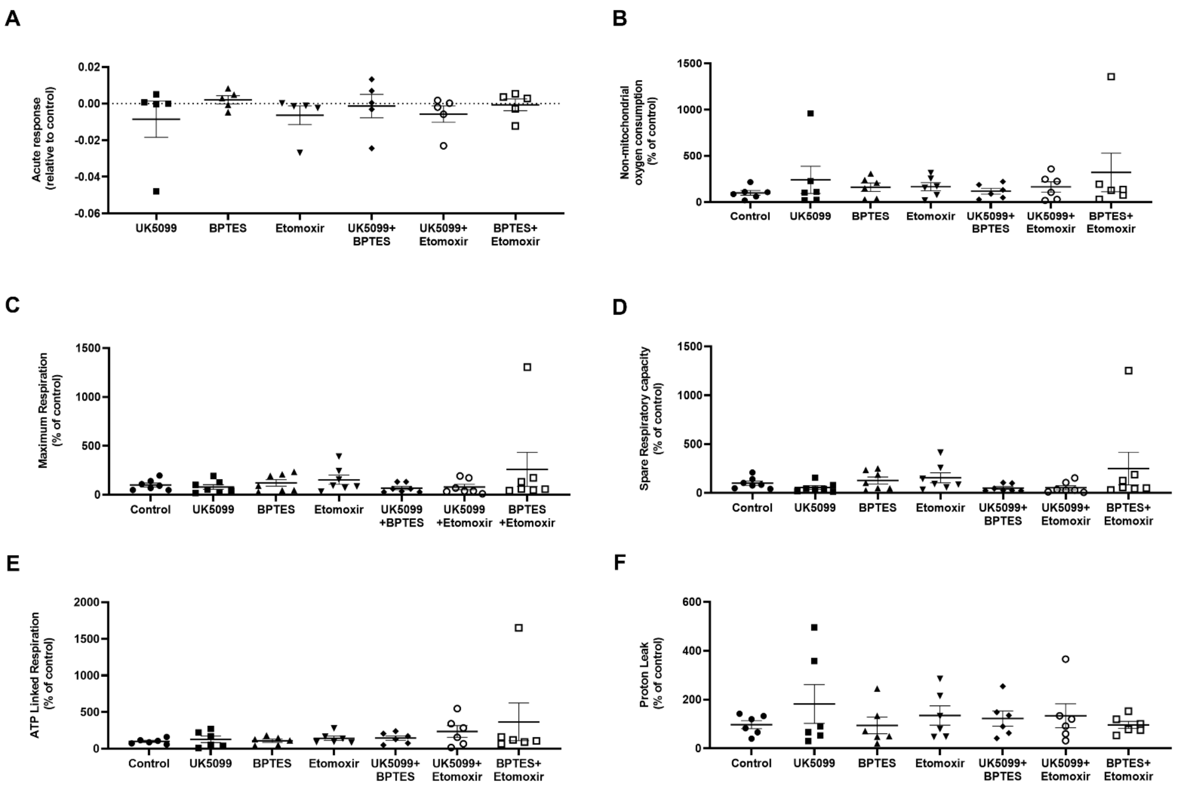

Figure S2. Influence of the inhibitors in (A) basal respiration (acute response); (B) non-mitochondrial oxygen consumption; (C) Maximum respiration oxygen consumption; (D) Spare respiratory capacity; (E) Proton Leak and (F) ATP link respiration in the VAT of patients with a class 3 obesity. Except for the acute response, all values are normalized to the control group.

Table S1. Correlation between Age (years) and visceral adipose tissue respiration parameters.

Parameter	Correlation (R value)	p value
Total Basal Oxygen Consumption	0.01	ns
Mitochondrial Basal Respiration	0.20	ns
Non-mitochondrial oxygen consumption	0.02	ns
ATP Link Respiration	0.31	ns
Proton Leak	0.28	ns
Maximum Respiration	0.21	ns
Spare Respiratory Capacity	0.13	ns

Table S2. Respiratory parameters calculation formulas.

**Assay parameter**	**Meaning/Equation**
Total Basal Oxygen Consumption	Last measure before the injection of the metabolic pathways` inhibitors
Non-mitochondrial oxygen consumption	Minimum rate measurement after Rotenone/Antimycin A injection
Mitochondrial Basal Respiration	(Last rate measurement before any injection) – (non-mitochondrial oxygen consumption)
ATP linked respiration	(Last rate measurement before Oligomycin injection) – (Minimum rate measurement after oligomycin injection)
Acute response	(Last rate measurement before oligomycin injection) – (Last rate measurement before acute inhibitor injection)
Maximum respiration	(Maximum rate measurement after FCCP injection) – (non-mitochondrial oxygen consumption)
Spare respiratory capacity	(Maximum respiration) – (Basal Respiration)
Proton leak	(Minimum rate measurement after oligomycin injection) – (non-mitochondrial oxygen consumption)

Table S3 Primers' sequences

Gene	Primer Sequence (Forward/Reverse)	T annealing (°C)	Size (bp)
**ob**	TCTTGTCCCCTCTTGACCCA<br>AGCTTGTGTTGCTGGGAGTT	52.60	183
**CPT1A**	GCAGCGTTCTTTGTGACGTT<br>AGGAGTGTTCAGCGTTGAGG	51.86	184
**SLC1A5**	TCTGGCTGGTAACCGCTACT<br>GAGTTTCTCTGGGTGGGAGC	52.81	107
**GLS1**	TCTACAGGATTGCGAACGTCT<br>CTTTGTCTAGCATGACACCATCT	50.31	100
**MPC1**	ATTTGCCTACAAGGTACAGCC<br>AGTCATCTCGTGTTTGATAAGCC	50.05	109
**SLC2A4**	TCCACCAACAACACCGAGAC<br>CGACCAGCATCTTCGAGACA	52.20	117
**RPII**	CTTCACGGTGCTGGGCATT<br>GTGCGGCTGCTTCCATAA	52.20	239
**LDHA**	CGACCGCCCGACGTG<br>TAAGGAAAAGGCTGCCATGTTG	52.94	264
**FABP4**	GTAGGAGTGGGCTTTGCCAC<br>ACGCATTCCACCACCAGTTT	53.01	287
**β-actin**	CCAACCGCGAGAAGATGA<br>CCAGAGGCGTACAGGGATAG	53.10	97

CPT1a – Carnitine Palmitoyl Transferase 1a; *FABP4* - Fatty Acid Binding-Protein 4; *GLS1* – Glutaminase 1; *LDHA* - Lactate Dehydrogenase A; *MPC1* – Mitochondrial Pyruvate Carrier 1; *ob* – Leptin; qRT-PCR - quantitative Real-Time Polymerase Chain reaction; *RPII* - RNA polymerase II; *SLC1A5* – Neutral amino acid transporter; *SLC2A4* – Glucose transporter 4; T – temperature

Table S4. - The three-step cycling qRT-PCR program used

Step	Tannealing (°C)	Time	N° of cycles
**Ativation**	95	2 min	1
**Desnaturation**	95	5 s	**35 cycles** (*CPT1a*, *GLS1*, *MPC1*)<br>**37 cycles** (*SLC1A5*)<br>**40 cycles** (*β-actin*, *RPII*, *FABP4*, *LDHA*)<br>**41 cycles** (*SLC2A4*)<br>**45 cycles** (*ob*)
**Annealing**	**50ºC** (*β-actin*, *FABP4*, *LDHA* and *RPII*)<br>**56ºC** (*CPT1a*, *GLS1*, *MPC1*)<br>**58ºC** (*SLC2A4* and *SLC1A5*)<br>**60ºC** (*ob*)	30 s	
**Extension**	72	1 min	
**Final Desnaturation**	72	7 min	1

CPT1a – Carnitine Palmitoyl Transferase 1a; *FABP4* - Fatty Acid Binding-Protein 4; *GLS1* – Glutaminase 1; *LDHA* - Lactate Dehydrogenase A; *MPC1* – Mitochondrial Pyruvate Carrier 1; *ob* – Leptin; qRT-PCR - quantitative Real-Time Polymerase Chain reaction; *RPII* - RNA polymerase II; *SLC1A5* – Neutral amino acid transporter; *SLC2A4* – Glucose transporter 4; T – temperature
